# Prevalence of Trypanosome Species in Cattle Near Ruma National Park, Lambwe Valley, Kenya: An Update From the Historical Focus for African Trypanosomosis

**DOI:** 10.3389/fvets.2021.750169

**Published:** 2021-11-02

**Authors:** Shewit Kalayou, Michael Nyang'anga Okal, Peter Otieno Odhiambo, Kawira Mathenge, Daniel Ochieng Gamba, Edward Kariuki, Francis McOdimba, Daniel Masiga

**Affiliations:** ^1^International Centre of Insect Physiology and Ecology (icipe), Nairobi, Kenya; ^2^Kenya Tsetse and Trypanosomiasis Eradication Council (KENTTEC), Nairobi, Kenya; ^3^Veterinary and Capture Service Department, Kenya Wildlife Service, Nairobi, Kenya; ^4^Department of Biological Sciences, Faculty of Science, Egerton University, Nairobi, Kenya

**Keywords:** bovine trypanosomosis, *T. brucei* subgroup, *T. congolense* savannah, *T. vivax*, wildlife-livestock interface, Lambwe valley, prevalence

## Abstract

The effective control of diseases in areas shared with wildlife depends on the validity of the epidemiologic parameters that guide interventions. Epidemiologic data on animal trypanosomosis in Lambwe valley are decades old, and the recent suspected outbreaks of the disease in the valley necessitate the urgent bridging of this data gap. This cross-sectional study estimated the prevalence of bovine trypanosomosis, identified risk factors, and investigated the occurrence of species with zoonotic potential in Lambwe valley. The area is ~324 km^2^, of which 120 km^2^ is the Ruma National Park. Blood was sampled from the jugular and marginal ear veins of 952 zebu cattle between December 2018 and February 2019 and tested for trypanosomes using the Buffy Coat Technique (BCT) and PCR-High-Resolution Melting (HRM) analysis of the 18S RNA locus. Risk factors for the disease were determined using logistic regression. The overall trypanosome prevalence was 11.0% by BCT [95% confidence interval (CI): 9.0–13.0] and 27.9% by PCR-HRM (95% CI: 25.1–30.8). With PCR-HRM as a reference, four species of trypanosomes were detected at prevalences of 12.7% for *T. congolense* savannah (95% CI: 10.6–14.8), 7.7% for *T. brucei brucei* (CI: 6.0–9.4), 8.7% for *T. vivax* (CI: 6.9–10.5), and 1.3% for *T. theileri* (CI: 0.6–2.0). About 2.4% of cattle had mixed infections (CI: 1.4–3.41). No human-infective trypanosomes were found. Infections clustered across villages but were not associated with animal age, sex, herd size, and distance from the park. Approximately 85% of infections occurred within 2 km of the park. These findings add to evidence that previous interventions eliminated human trypanosomosis but not bovine trypanosomosis. Risk-tailored intervention within 2 km of Ruma Park, especially in the north and south ends, coupled with stringent screening with molecular tools, could significantly reduce bovine trypanosomosis.

## Introduction

Since time immemorial, several countries in sub-Saharan Africa have struggled with the debilitating burden of human and animal trypanosomosis. This group of diseases caused by protozoan parasites of the genus *Trypanosoma* and transmitted primarily by tsetse flies limits human health and livestock production in more than 32 countries of sub-Saharan Africa (1). Several interventions with varied intensity, frequency, approach, and success rates (2, 3) have been implemented in different regions, with significant progress in controlling the disease in humans and animals. With <1,000 cases of Human African Trypanosomosis (HAT) now reported in a year, the focus is on eliminating the disease by 2030 (4). On the contrary, Animal African Trypanosomomosis (AAT) is still widespread, occurring in more than 10 million km^2^ of land where it kills about 3 million cattle every year (1). Approximately one-quarter of Kenya is tsetse infested and endemic for AAT (5). The impact of AAT is disproportionate; persons living near wildernesses, wildlife protectorates, and other interfaces with wildlife, such as that of the Ruma National Park (RNP) in Lambwe valley, bear the brunt of the disease.

Lambwe valley epitomizes the struggle against HAT and AAT (6, 7). Evidence suggests that several trypanosome species circulated long before the first unnamed settler arrived in 1951 (8). However, it was the detection of the zoonotic *Trypanosoma brucei rhodensiense* in the valley in 1959 (9) and the consequent steep rise in cases of sleeping sickness (8) that necessitated studies aimed at characterizing the epidemiology of trypanosomes in the valley in attempts to eradicate the diseases. The major tsetse population that infests Lambwe valley, *Glossina pallidipes*, is resilient and has repeatedly recovered from attempts at eradication in the last half a century. Between 1968 and 1971, bushes were cleared, and residual dieldrin was applied to the valley with aerial and ground spraying (8). This was repeated from 1981, this time with endosulfan applied in addition to dieldrin. As a result, tsetse populations reduced by 99.9% by 1989. Both times, the flies and, therefore, the disease recovered to pre-intervention levels presumably because of technical difficulties and complexities of dealing with a large, entrenched tsetse population living in environmentally optimal conditions (8). Over time, ambitions for tsetse eradication were replaced with interventions to control the vector (10). Active multiagency tsetse control continued until 1998 (8).

The lack of explicit information for the prevalence of pathogens, especially in the wildlife-livestock-human interfaces, is a major drawback for effective control of AAT (11). Consequently, the recently defined progressive control pathway for AAT advocates for enhanced research to understand the risks of trypanosomosis to guide the selection of priority intervention areas as the first step toward effective control (12). For Lambwe valley, epidemiologic studies investigating the prevalence of trypanosomes are scanty and decades-old (7, 13). A proactive approach to updating the epidemiology of AAT is therefore critical in designing interventions in the future. Notably, available HAT data are passively and retrospectively collected from clinics and cannot sufficiently determine the state of disease; studies are required to affirm the absence of livestock reservoirs for the causative agents for HAT.

Sensitive and specific tools are critical for accurately diagnosing trypanosomes and describing the prevalence. Traditionally, microscopy has been used for trypanosome detection in the field (14). This method involves blood concentration in a microhematocrit capillary tube followed by an examination of buffy coats. Despite being simple and inexpensive, the method is unreliable for immature forms and less sensitive in samples with low parasitemia and trypanosome co-infections (15). The advent of polymerase chain reaction (PCR) and the increased development of molecular markers that target different regions of the trypanosome genome improved the sensitivity significantly (16). Some of the molecular targets that are currently used to detect Trypanosome species include (i) the internal transcribed spacer (ITS) because it is highly conserved and has different size variability, which allows trypanosome identification and sub-grouping (16); (ii) the glyceraldehyde phosphate dehydrogenase (GAPDH), a housekeeping gene, which is more convenient for extensive phylogenetic analysis and, thus, a better marker for novel species identification (17); and (iii) the small subunit (SSU), 18S, and the large subunit (LSU), 28S rRNA genes (18). Historically, studies in Lambwe valley were based on microscopy (7, 13).

In December of 2019, we responded to distress calls from the county government of Homa Bay, smallholders in Lambwe valley, and a local non-governmental organization citing a suspected outbreak of AAT and possible HAT cases. The main symptoms in cattle reported included staring coat, swollen lymph nodes, lethargy, lacrimation, and emaciation. These symptoms are non-specific and also associated with helminthosis, malnutrition, anaplasmosis, and other tick-borne diseases (19). We implemented a large study investigating the molecular epidemiology of tick-borne pathogens and found a 78.5% prevalence of tick-borne pathogens (20), including a novel *Anaplasma* spp. with unknown pathogenicity to livestock or humans. By combining microscopic and PCR-High-Resolution Melting (HRM) analysis (21), this cross-sectional study aimed to assess the agreement of these assays, determine the prevalence of bovine trypanosomosis, identify risk factors, and investigate the occurrence of potentially zoonotic species in cattle in the historical focus of Lambwe valley.

## Materials and Methods

### Study Area and Setting

**Figure 1:**
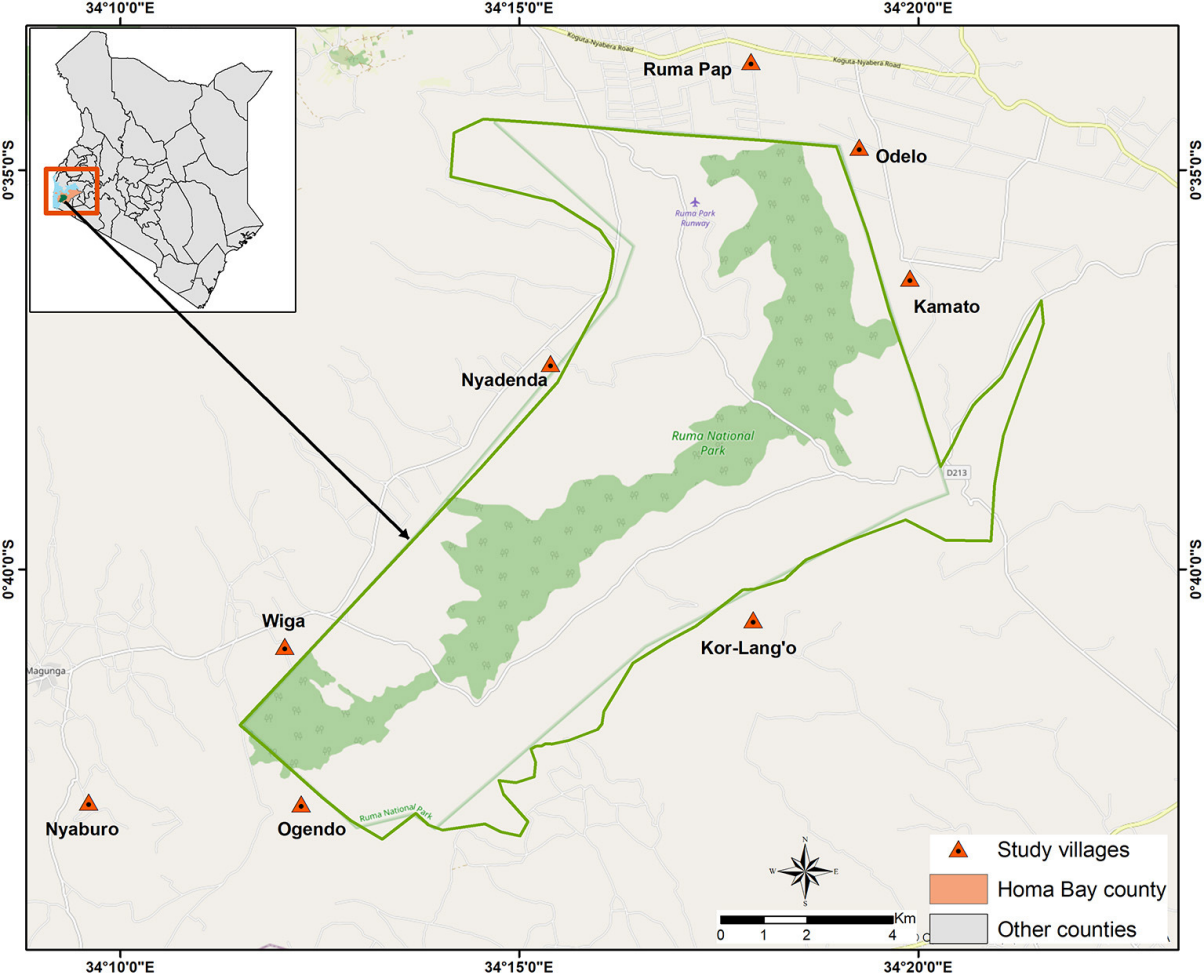
Map of the study area indicating villages where cattle were sampled.

The study was implemented in Lambwe Valley, in village clusters around the RNP (Figure 1). Lambwe Valley (latitude 0° 38′ 35.52″ S, longitude 34° 16′ 48″ E) is located south of the equator in Homa Bay County, Kenya at 1,200–1,600 m above sea level. The valley extends over 350 km^2^ and hosts RNP, a wildlife protectorate of 120 km^2^. The terrain consists of rolling grasslands with open woodland and thickets dominated by acacia and various grass species. The soil is predominantly black cotton, and the climate is hot and humid, with an average annual air temperature of 25°C. Rainfall is bimodal, peaking in April–June and September–November. The dry and hot periods lie between January and March (22). The community in Lambwe Valley practices subsistence farming, fishing, and animal husbandry. In the park, the primary grazing and browsing wild ruminant populations consist of roan antelope (*Hippotragus equinus langheldi*), Jackson's hartebeest (*Alcelaphus buselaphus Jacksonii*), oribi (*Ourebia ourebi*), and Rothschild giraffes (*Giraffa camelopardalis rothschildi*). The indigenous zebu cattle breed is the dominant domestic species, followed by small ruminants. Livestock grazes in the open fields extending to the park's fence, thus creating an interface for interaction between wildlife and livestock. The valley is infested by *G. pallidipes*, the major vector for African animal trypanosomosis. *G. fuscipes fuscipes* distribution is limited to the lakeshore and riverine forest habitat.

### Cattle Herds, Sample Size Estimation, and Sampling Method

**Figure 2:**
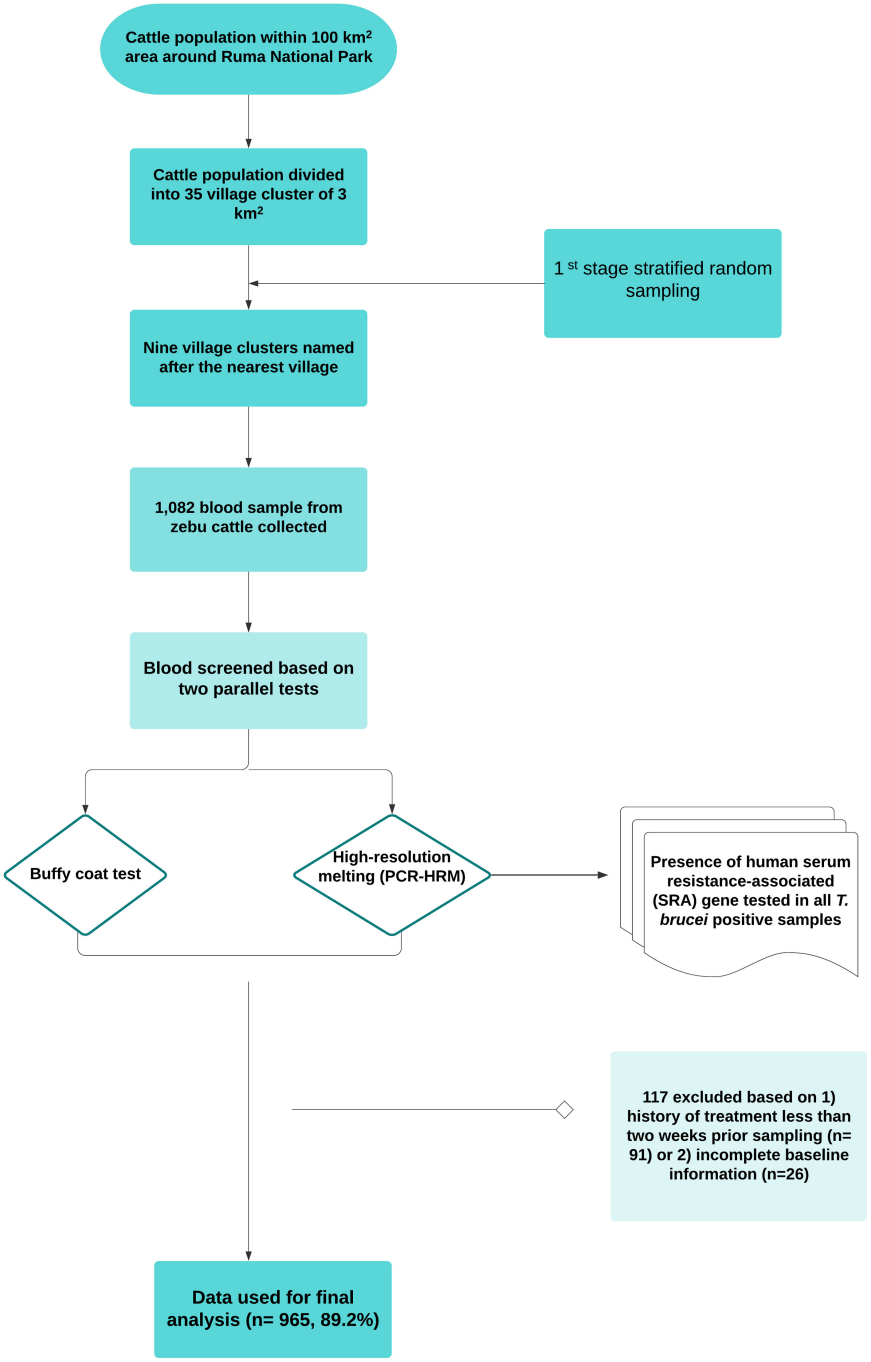
Workflow of the study design and sampling.

We used a cross-sectional design with stratified one-stage cluster sampling. Details of the design and setting were described in our previous study (20). Data were collected between December 2018 and February 2019. The source population was defined as all cattle reared in the wildlife–human–livestock interface of the Lambwe valley so that all zebu cattle within a 5-km radius from the park's fence were eligible for inclusion. The construction of a sampling frame was not possible due to the lack of reliable cattle demographic data. Therefore, all herds that voluntarily responded to a mobilization drive by the Kenya Tsetse and Trypanosomiasis Eradication Council were sampled. Administrative boundaries are often not epidemiologically meaningful for vector-borne diseases such as trypanosomosis. Considering this, we created a grid based on a 100-km^2^ area around the park (~1/3 of the valley) and divided the cattle population into 35 3-km^2^ grids, which later was named after the nearest village. Therefore, the primary sampling frame was a list of spatial clusters “villages” where herds of cattle are kept. Information on the total number of herds in the study area was missing, and therefore all herds were sampled. No animal was sampled within the park or where there was no human settlement. The workflow, study design, and sampling approach are outlined in Figure 2.

Indigenous zebu cattle that were at least 1 year old and managed in smallholder farms in the wildlife–livestock interface of the Lambwe Valley were considered eligible for the study. This age category is highly likely to interact with other herds or wildlife at watering points and during grazing, thus amenable to surveillance in the area. Sample size determination was based on one-stage cluster sampling using previously described methods (23–25).

The sample size to estimate the prevalence with a specified precision is given by:

*N* = gc = P(1−P) D/ [(SE)] ^∧^2 (Equation 1), where N is the sample size, P is the prevalence, D is the design effect, SE is the standard error of an estimated proportion P, g is the average number of animals sampled per cluster, and c the number of clusters sampled. The design effect was given by the next formula (23): D = 1 + (g– 1) ICC (Equation 2).

We restricted the number of sampled villages (clusters) to 25% (*c* = 9) of 35 village clusters. The selection of these villages was first made randomly. The final listing of villages was done after accounting for accessibility. We used an intra-cluster correlation (ICC) value of 0.13 for *Trypanosoma congolense* from previous studies (26). The ICC is a measure of homogeneity of clustered data. Considering the possibility to collect a maximum of 100 samples by a team of six people per day per cluster, D was calculated as 13.87 (Equation 2). Molecular prevalence information for trypanosomosis is limited in the study area. Therefore, the study assumed a prevalence (*P*) of 17% from a similar Maasai Steppe wildlife–livestock interface in Tanzania (27). With an expected prevalence of 17%, a cluster size of 9 and a desired precision of 5%, a total sample size of 783 animals was estimated. The study was announced to farmers for sensitization through local radio, accounting for a 15% non-response rate. To consider sample size for non-response rate:

*N* = (Sample size calculated)/(1 – non-response rate) (Equation 3). The above equations gave an estimated sample size of 921. A total of 1,082 blood samples were collected. However, 117 samples were excluded based on (1) history of treatment <2 weeks prior to sampling (*n* = 91) and (2) incomplete baseline information (*n* = 26). The final dataset consisted of a total of 952 cattle.

Epidemiological data on potential risk factors were collected from each sampled household using a questionnaire. The following variables were captured: sex, herd size, and village and homestead distance from the park's fence. Distance from the park was used as a conservative approach to identifying areas with greater risk for trypanosomosis. The position of the homestead was marked by GPS (Garmin GPS). Furthermore, herd husbandry practices including trypanocidal drug usage, sources of drug, treatment frequency, and personnel involved in the treatment of cases were also collected.

### Blood Collection and Processing

During the survey, two blood samples from each cattle were taken in the early morning. The specimen consisted of blood samples collected from (1) the marginal ear vein to determine the level of anemia level, examination of buffy coat, and the preparation of thin blood films; and (2) the jugular vein blood for the molecular detection of trypanosomes by PCR-HRM.

Each animal was assessed for anemia by measuring packed cell volume (PCV) using the micro-hematocrit method. Briefly, capillary blood was drawn into EDTA microhematocrit tubes and centrifuged at 13,000 *g* for 10 min. Then, PCV levels were calculated using the Hawksley hematocrit reader.

Detection of trypanosome species was performed by two parallel tests, buffy coat technique (14) and PCR-HRM (28). Morphological identification of *Trypanosomes* was conducted by microscopic examination of Giemsa-stained fixed thin blood smear. For molecular detection of trypanosomes, about 4 ml of blood was collected from the jugular vein of each cattle using sterile vacutainer needles and EDTA vacutainers. A unique animal's number identified each tube. Blood in the EDTA vacutainers was transferred into uniquely labeled cryovials, stored in liquid nitrogen, and transported to *icipe's* Martin Lüscher-Emerging Infectious Diseases (ML-EID) Laboratory for molecular analysis.

### Real-Time PCR and High-Resolution Melting Analysis

DNA was extracted from whole blood samples using the Bioline Isolate II genomic DNA kit (Meridian Life Science, Memphis, USA) as described by the manufacturer. A single-plex PCR was performed to screen for the presence of *Trypanosoma* spp. using 18S rRNA trypanosome-specific primers, as shown in Table 1. PCR was carried out in a 10-μl volume consisting of 1 μl of the extracted DNA template, 2 μl 5 × H.O.T. FIREPol® EvaGreen H.R.M. Mix (Solis BioDyne, Tartu, Estonia), 0.5 μl of each 10 μM forward and reverse primers for the respective genus-specific reactions, and 6 μl of PCR water. The PCR amplification assays were done in QuantStudio 3 (Applied Biosystems) HRM capable thermocycler. The amplification condition involved an initial activation of the polymerase enzyme at 95°C for 15 min, followed by 40 cycles of denaturation at 94°C for 30 s, annealing at 62°C for 30 s, extension at 72°C for 30 s, and final extension steps at 72°C for 10 min. The fluorescence data were captured at the end of every cycle at the extension stage on the green channel (excitation at 470 nm and emission at 510 nm detection spectrum). HRM analysis was performed post PCR where the PCR amplicons were gradually dissociated at 0.1°C temperature increments from 75 to 97°C with fluorescence acquisition data captured every 2 s. The unknown PCR samples were identified by comparing the different melting profiles with the positive controls using the HRM analysis software version 2.1.0 (Qiagen).

**Table 1:** List of primers used for trypanosome species and SRA gene identification.

**Target gene**	**Primer**	**Primer sequence**	**Trypanosome species**	**Amplicon size (bp)**	**References**
18S	18S-3F	GACCRTTGTAGTCCACACTG	*T. congolense*	233	(18)
	18S-4R	CCCCCTGAGACTGTAACCTC	*T. brucei*	234	
			*T. vivax*	199	
TBR	TBR-1	CGAATGAATATTAAACAATGCGCAGT	*T. brucei* subspecies	177	(29)
	TBR-2	AGAACCATTTATTAGCTTTGTTGC			
SRA	SRA-A	GACAACAAGTACCTTGGCGC	*T.b. rhodesiense*	460	(30)
	SRA-E	TACTGTTGTTGTACCGCCGC			
SRA	B537	CCATGGCCTTTGACGAAGAGCCCG	*T.b. rhodesiense*	743	(31)
	B538	CTCGAGTTTGCTTTTCTGTATTTTTCCC			

### SRA PCR for Detection of *T.b. rhodesiense* in Cattle

#### PCR Confirmation of *T. brucei* Subspecies

To ascertain whether the HRM positive samples were indeed *T. brucei* subspecies of trypanosome, *T. brucei* 18S PCR-HRM positive samples were amplified by TBR1/2 primers specific for *T. brucei* subspecies (Table 1). PCR amplification was carried out in 10 μl volume containing 3.0 μl of nuclease-free water, 5.0 μl of HotStarTaq Master Mix (Qiagen), 0.5 μl of 10-μm primers, and 1.0 μl of DNA template. Negative control was included to confirm the absence of contamination. The amplification was carried out in ProFlex PCR System (Applied Biosystems) with an initial denaturation step at 95°C for 15 min followed by 35 cycles of denaturation at 95°C for 1 min, annealing at 55°C for 1 min, and extension at 72°C for 1 min and final elongation at 72°C for 5 min. The PCR amplicons were visualized in 2.0% w/v agarose gel stained with 5.0 μl of 10 mg/ml of ethidium bromide.

#### SRA PCR for Detection of *T.b. rhodesiense* in Cattle

Amplification of the SRA gene was conducted using the primers B537/B538 and SRA-A/SRA-E (Table 1) specific for *T.b. rhodesiense*. The amplification was performed in a 10-μl reaction volume containing 3.0 μl of nuclease-free water, 5.0 μl of HotStarTaq Master Mix (Qiagen), 0.5 μl of 10-μm primers, and 1.0 μl of purified DNA template. The amplification profile using SRA-A/SRA-E was as follows: initial denaturation step at 95°C for 15 min followed by 45 cycles of denaturation at 95°C for 1 min, annealing at 68°C for 1 min and extension at 72°C for 1 min, and final elongation at 72°C for 10 min. Detection of *T.b. rhodesiense* SRA gene using the B537/B538 primer set involved an initial denaturation step at 95°C for 15 min and 10 cycles at 94°C for 20 s, 55°C for 30 s, and 72°C for 1 min followed by 30 cycles at 95°C for 20 s and 64°C for 30 s, and extension at 72°C for 1-min extension time was increased by 2 s per cycle and final elongation at 72°C for 10 min. All amplification was performed in ProFlex PCR System (Applied Biosystems). The PCR amplicons were visualized in 2% w/v agarose gel stained with 5.0 μl of 10 mg/ml of ethidium bromide.

### Data Management and Analysis

A database consisting of a questionnaire and molecular data was established in Microsoft. Excel (Microsoft® Excel, Washington). Individual- and herd-level information, including animal sex, age, animal weight, anemia status, herd size, village name, the distance of homestead from the park's fence, drug use (type and frequency), and infection status, was recorded. Distance of homestead from the park's fence was measured using a handheld GPS device. Age of cattle was categorized into ≤ 2 (calves and weaners) and >2 (adults) years and herd size was categorized into small (<10 head of cattle) or medium (≥10 head of cattle). Anemia status was categorized into anemic (PCV ≤ 24) and normal (PCV > 24). After checking and variable coding, data were transferred to SPSS version 25 (I.B.M. Corp, Armonk, NY) for descriptive statistics and univariable regression. The degree of agreement between BCT and PCR-HRM techniques was determined in matching blood samples by calculating kappa (κ) values in SPSS 25 suite. κ values express the agreement beyond chance and were interpreted according to Altman (32).

The prevalence of each trypanosome species was calculated by dividing the number of positive samples by the total animal sample during the study period. Overall and species-level prevalence and 95% confidence intervals were calculated as Wilson Score intervals. The relationship between the presence of trypanosomes and putative risk factors was modeled using univariable logistic regression models. The odds ratio with its 95% confidence interval was used as a summary statistic.

## Results

### Parasitological and Molecular Detection of Trypanosome Infection

**Figure 3:**
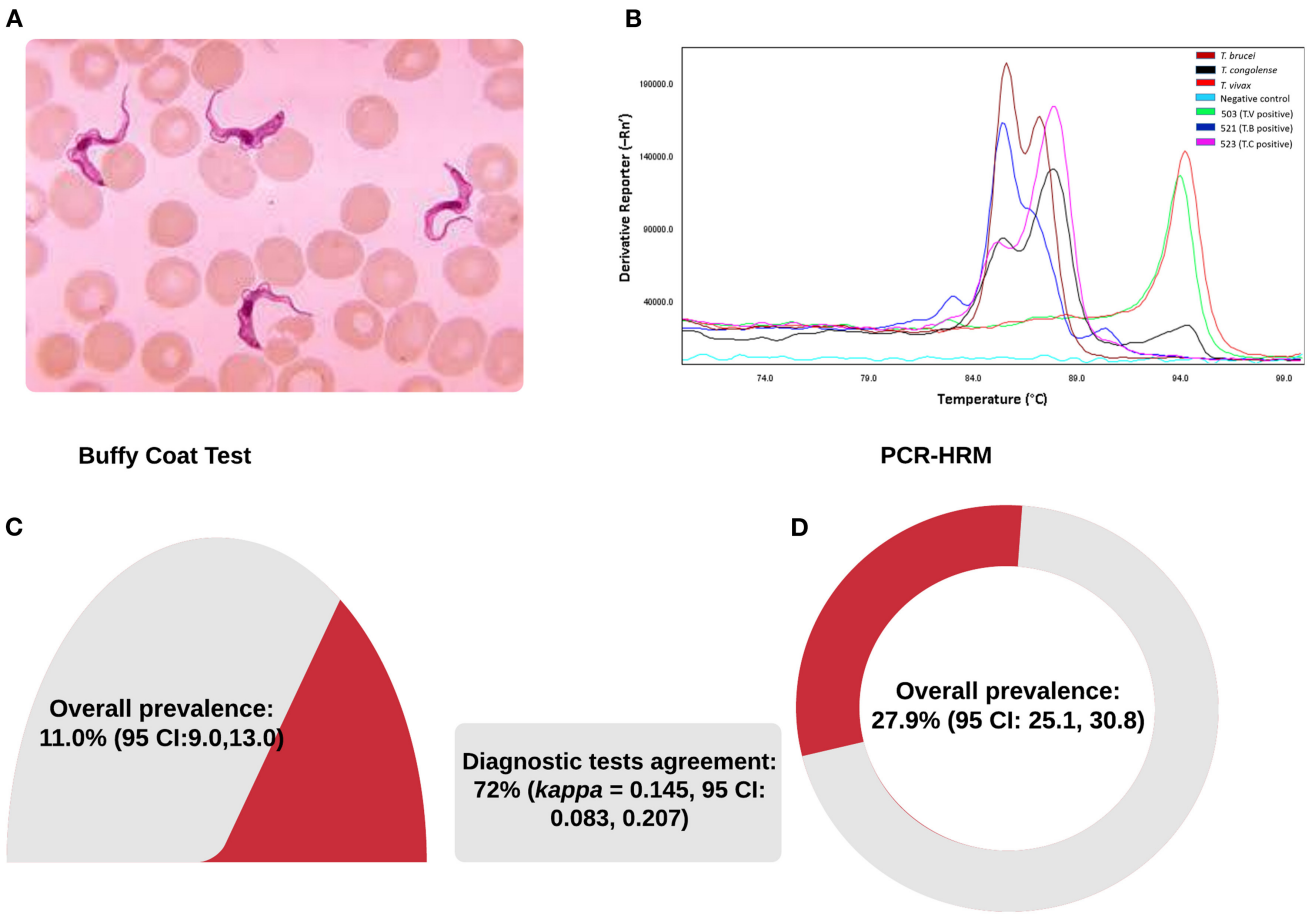
Trypanosome species as identified by microscopy **(A)**, PCR-HRM **(B)**, and prevalence **(C,D)**.

A total of 952 blood samples were tested in nine village clusters parallelly by BCT microscopy and PCR-HRM. The samples were all local zebu cattle from 164 herds with an average herd size of seven and were maintained under a free-range grazing system. The distance range between the chosen village clusters and the RNP fence was between 0 (homesteads within the park's fence) and 4,461 m (median 806 m). Parasitological and molecular test results are summarized in Figure 3 and Table 2. Four trypanosome species, *T. brucei brucei, T. congolense, T. theileri*, and *T. vivax*, were present in the study area. The overall trypanosome infection prevalence using BCT and PCR-HRM tests was 11.0% (95% CI: 9.0–13.0) and 27.9% (95% CI: 25.1–30.8), respectively (Figure 3, Table 2).

**Table 2:** Comparison of diagnostic test results obtained by microscopic (BCT) and molecular method (PCR-HRM).

**Microscopy (BCT)**	**PCR-HRM**							**Total (%)**
	**Tb**	**Tc**	**Tt**	**Tv**	**Tb/Tv**	**Tc/Tv**	**Negatives**	
Tb	1^*^	1	1	0	0	0	1	4 (0.4)
Tc	8	27^*^	0	6	4	0	32	77 (8.1)
Tt	0	0	0	0	0	0	1	1 (0.1)
Tv	1	1	0	1^*^	0	0	16	19 (2.0)
Tb/Tv	0	0	0	0	0	0	0	0 (0.0)
Tc/Tv	0	1	0	0	0	0	2	3 (0.3)
Negatives	59	91	11	72	15	4	633^*^	847 (89.0)
Total (%)	54 (5.7)	117 (12.3)	12 (1.3)	60(6.3)	19 (2.0)	4 (0.4)	686 (72.1)	952

*Tb, Trypanosoma brucei; Tc, T. congolense; Tt, T. theileri; Tv, T. vivax; Tb/Tv, mixed infection of T. brucei and T. vivax; Tc/Tv, mixed infection of T. congolense and T. vivax*.

* *Number of positive or negative samples detected by both BCT and PCR-HRM methods*.

BCT detection showed 105 animals infected by one or more species of trypanosomes, and *T. congolense* was the most prevalent species (Table 2). PCR-HRM detection showed 266 infected animals. Like the BCT method, the most prevalent trypanosome species by PCR-HRM was *T. congolense* (12.7%, 95% CI: 10.6–14.8), followed by *T. vivax* (8.7%, 95% CI: 6.9–10.5), and *T. brucei brucei* (7.7%, 95% CI: 6.0–9.4). Infection with single trypanosome species accounted for most infections (243/266 by PCR-HRM and 102/105 by BCT method). The prevalence of mixed infection was 2.3% by PCR-HRM and was significantly higher than the BCT method (0.3%). More than 82% (19 out of 23) of mixed infection identified by PCR-HRM was due to *T. vivax* and *T. brucei brucei*. Four samples were co-infected by *T. congolense* and *T. vivax*. All the three mixed infections detected by BCT microscopy were due to *T. congolense* and *T. vivax*.

Performance of the BCT and PCR-HRM was assessed in 952 matching blood samples. The degree of agreement was found to be poor (72%, Cohen's *k* = 0.145, 95% CI: 0.083–0.207). Comparing PCR-HRM method as a reference, BCT microscopy showed poor sensitivity to detect all species. Nonetheless, the specificity was well above 90% (Table 3). BCT has a sensitivity of 20% (95% CI: 15–25%), a specificity of 92% (95% CI: 90–94), a positive predictive value of 49.5% (95% CI: 44–56), and negative predictive value of 74.5% (95% CI: 74–75). *Trypanosoma vivax* and *T. brucei brucei* were the species most missed by BCT microscopy examination.

**Table 3:** Prevalence of Trypanosome species in naturally infected cattle in Lambwe valley, and the sensitivity and specificity of BCT, using PCR-HRM as a reference test.

**Trypanosoma**		**Point estimates**	**95% CI**	
			**Lower**	**Upper**
*T. brucei*	Prevalence	7.7	6.0	9.4
	Sensitivity	0.014	0.0003	0.074
	Specificity	0.997	0.99	0.999
*T. congolense*	Prevalence	12.7	10.6	14.8
	Sensitivity	0.231	0.16	0.32
	Specificity	0.94	0.92	0.95
*T. vivax*	Prevalence	8.7	6.9	10.5
	Sensitivity	0.012	0.0003	0.065
	Specificity	0.976	0.963	0.985
*T. theileri*	Prevalence	1.3	0.6	2.0
	Sensitivity	0	0	0.27
	Specificity	0.999	0.99	1
Overall	Prevalence	27.9	25.1	30.8
	Sensitivity	0.2	0.15	0.25
	Specificity	0.92	0.90	0.94
	PPV^*^	0.495	0.44	0.56
	NPV^*^	0.747	0.74	0.75

* *PPV, positive predictive value; NPV, negative predictive value*.

SRA-PCR analysis showed that none of the *T. brucei* subspecies positive samples was positive for the human-infective trypanosome species (*T.b. rhodesiense*).

Presence of trypanosomes was associated with anemia level (χ^2^ = 4.563, *df* = 1, *p* = 0.038). While infection with the *T. vivax* and *T. brucei* subgroup was not associated with PCV level, 44% of *T. congolense* infected cattle were below 24%, and this proportion was significantly higher than negative cattle (28.5%, χ^2^ = 11.417, *p* = 0.001) (Supplementary Table 1).

### Association of Putative Risk Factors With Trypanosome Infection

Table 4 presents the results from the univariable logistic regression analysis of the association between individual-level exposure and trypanosome infection status.

**Table 4:** Risk factors associated with trypanosome infection status using PCR-HRM as a reference test.

**Risk factors**	**Category level**	***n* ^**a**^**	**Overall prevalence (95% CI)**	***T. congolense***		***T. vivax***		***T. brucei***	
				**Prevalence (%)**	**95% CI**	**Prevalence (%)**	**95% CI**	**Prevalence (%)**	**95% CI**
Village	Kor-Lango	56	10.7 (5.0, 21.5)	1.8	0.3, 9.4	3.6	0.9, 12	1.8	0.3, 9.4
	Odelo	68	19.1 (11.5, 30.0)	7.4	3.2, 16.1	4.4	1.5, 12.2	7.4	3.2, 16.1
	Nyadenda	200	23.0 (17.7, 29.3)	15.0	10.7, 20.6	2.5	1.1, 5.7	4.5	2.4, 8.3
	Gendo	68	23.5 (15.0, 34.9)	8.8	4.1, 17.9	10.3	5.1, 19.8	1.5	0.3, 7.9
	Wiga	68	23.5 (15.0, 34.9)	8.8	4.1, 17.9	5.9	2.3, 14.2	1.5	0.3, 7.9
	Kamato	190	27.9 (22.0, 36.7)	16.3	11.7, 22.2	3.7	1.8, 7.4	8.4	5.3, 13.2
	Nyaburo	115	29.6 (22.0, 38.5)	10.4	6.1, 17.4	12.2	7.4, 19.4	13.0	8.1, 20.4
	Ruma Pap	122	33.6 (25.8, 42.4)	12.3	7.6, 19.3	20.5	14.3, 28.5	10.7	6.3, 17.4
	Ogendo	65	63.1 (50.9, 73.8)	23.1	14.5, 34.6	24.6	15.8, 36.3	18.5	10.9, 29.6
Herd size
	Small	513	30.2 (26.4, 34.3)	13.5	10.8, 16.7	10.3	7.9, 13.3	7.8	5.8, 10.4
	Medium	111	25.3 (21.5, 29.6)	11.8	9.2, 15.2	6.8	4.8, 9.6	7.5	5.4, 10.4
Sex
	Male	461	26.9 (23.1, 31.1)	12.6	9.9, 15.9	7.8	5.7, 10.6	6.5	4.6, 9.1
	Female	491	28.9 (25.1, 33.1)	12.8	10.2, 16.1	9.6	7.3, 12.5	8.8	6.6, 11.6
Age
	Calves and weaners	158	24.1 (18.1, 31.3)	8.2	4.9, 13.6	10.1	6.3, 15.8	8.2	4.9, 13.6
	Adults	792	28.8 (25.7, 32.0)	13.6	11.4, 16.2	8.5	6.7, 10.6	7.6	5.9, 9.6
Distance
	≤ 1 km	525	28.4 (24.7, 32.4)	14.3	11.6, 17.5	8.4	6.3, 11.1	6.5	4.7, 8.9
	>1 km	427	27.4 (23.4, 31.8)	10.8	8.2, 14.1	9.1	6.8, 12.2	9.1	6.8, 12.2

Overall, the prevalence of trypanosomosis was higher in small herds (30.2%), female cattle (28.9%), adult cattle (28.8%), and in homesteads <1 km from the park (28.4%). However, the association was not statistically significant.

**Figure 4:**
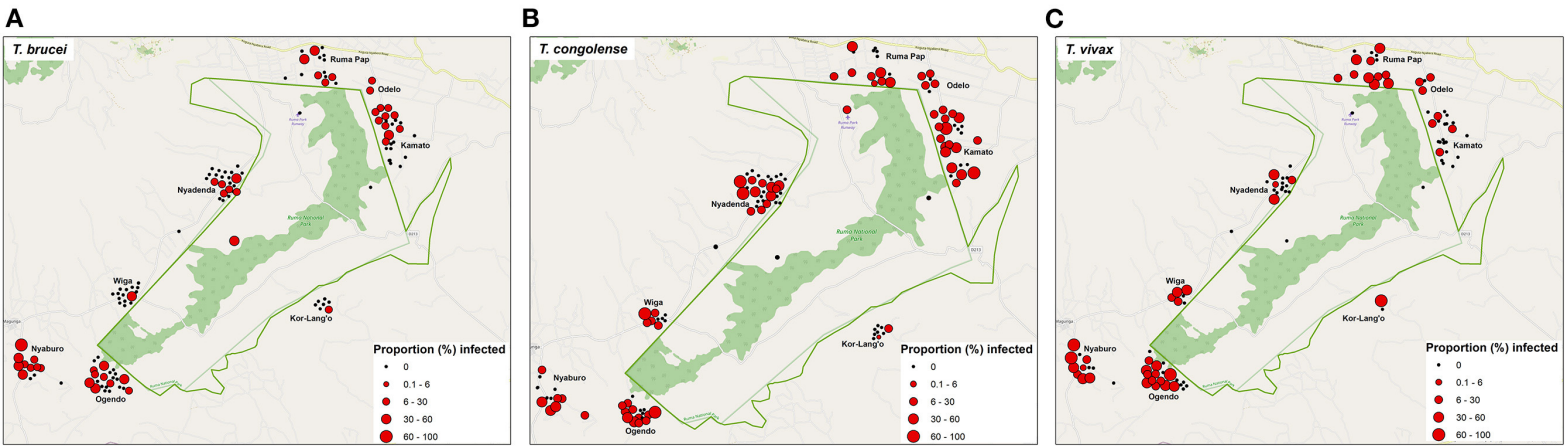
Distribution map showing the pathogenic trypanosome species detected and overlay with the proportion of infected cattle in each homestead **(A–C)** as tested by PCR method.

The prevalence of trypanosomes varied between the village clusters significantly (*p* < 0.001). The distribution of the three pathogenic trypanosome species across the spatially clustered villages is as shown in Figure 4. The overall trypanosomosis prevalence ranged from 10.9 to 63.1%. The four villages with the higher trypanosome prevalence were Ogendo (63%), Ruma Pap (33.6%), Nyaburo (29.6%), and Kamato (27.9%). The lowest infection rate was found in Kor-Lango (10.7%).

The risk factor analysis did not include *T. theileri* due to the availability of sufficient data to perform a risk factor analysis.

## Discussion

We found that the prevalence of bovine trypanosomosis in Lambwe valley was high and comparable to previous years. The BCT estimate of 11% prevalence is within the range of earlier studies and the historical data for the area. Robson and Ashkar estimated a prevalence rate of 17% in 1972 (7). Twenty years later, following periods of tsetse control and increasing human habitation of the valley (8), Nesbitt et al. (13) reported a lower rate for AAT at 5.6% in 1991. The differences in prevalence over time could be due to varied approaches to sampling, the effect of interventions, or just the characteristic flare-up and quiescence of trypanosomosis in the Lambwe valley (8).

With a molecular prevalence of 27.9%, our results demonstrated that cattle around RNP remain at a relatively high risk of AAT. The risk for AAT around wildlife interfaces in Kenya is varied, with the highest molecular prevalence estimated at 33.9% recorded in smallholder farms at the interface of the Shimba Hills (33). A comparatively lower molecular prevalence of 17.2% was reported for the Maasai Steppe in Tanzania (27). However, the prevalence rates for bovine trypanosomosis in the Lambwe valley at the time of this study were in agreement with previously recorded rates for the area (7, 13) or that of other regions endemic for the disease in Kenya (34) and in different areas (35–37). The minor discrepancies in prevalence between studies are likely associated with differences in sampling design, diagnostic method, location, and ecological factors such as wild host and farming practices.

Consistent with previous studies, the present work identified three trypanosome species that adversely affect cattle and the relatively little pathogenic species, *T. theileri*. Of these, *T. congolense* was the most important and accounted for nearly half of all infections, 45.5% (121/266). Similar to previous reports from Lambwe, the second-highest infections were of *T. vivax* (31.2%, 83/266) and after that, *T. brucei brucei* (27.4%, 73/266) (7, 13). The role of cattle and wildlife in sustaining the sylvatic cycle of *T. brucei rhodesiense*, especially in wildlife–livestock interfaces, is well-established (6, 11, 38–40). In a survey on small ruminants and cattle in Ngorongoro conservation in Tanzania, Ruiz et al. (39) reported that 2.1% of cattle had *T. brucei rhodesiense*. While Robson and Ashkar (7) recorded a 10.5% prevalence of *T.b. rhodesiense* in cattle in the Lambwe valley in 1972, we found no evidence of the human infective pathogen during this study. This finding indicates that the livestock reservoir for *T. brucei rhodesiense* around RNP, if any, is minimal and adds evidence that the pathogen may have been eliminated from the Lambwe valley by past interventions that included intensified proactive testing and treating of human reservoirs.

This study showed considerable heterogeneity in the villages with evidence of geographical clustering of trypanosomosis. With a prevalence of 10.7%, the least odds of infection were found in Kor-Lango. Though not investigated in this study, it might be attributable to variability in the local tsetse ecology. In a study implemented in Nguruman, Southwest Kenya, Odulaja et al. (41) showed that *G. pallidipes*, the same tsetse species that historically infests Lambwe valley, was highly aggregated at a scale of 4 km (41). Microaggregation of tsetse fly populations has been shown to correlate with the spatial prevalence of trypanosomosis (41). Kor-Lango is located in the southeastern part of the park and more than 4 km of other study villages.

We found that 85% of all infections occurred <2 km from RNP. However, analyses of risk factors did not associate the distance of homesteads from the park with differences in infection rates. Wildlife-protected areas are known to increase the risk for tsetse bites and trypanosomal disease (8). Indeed, previous studies in Lambwe have attributed the endemicity of African trypanosomosis to the presence of wild animal reservoir for trypanosomes and a ready source of blood meals for vector populations (8). The household locations used in risk factor analysis do not reflect cattle movement relative to the park during tsetse pick biting hours, and this might have weakened the association if grazing lands were different from the location of homesteads. Human occupation of Lambwe valley has steadily increased with consequent tsetse habitats destruction beyond protected area (42). The farmers who responded to this sampling effort lived near RNP and within the daily flight range of tsetse (43). It is plausible that this bias in the drive of farmers responding to the call reflects their disproportionate exposure and higher risk for AAT. The putative risk factors of animal age, sex, and herd size showed no significant association with the trypanosome species infection.

Our findings show that the BCT microscopic method can underestimate the detection of trypanosomes in cattle. We reported a more than 2-fold lower prevalence of AAT with BCT than PCR-HRM. Furthermore, using microscopy, we missed several infections with *T. vivax, T. brucei brucei*, and mixed trypanosome species infections when screening with BCT. For its relative affordability and ease of use, especially in field conditions, microscopy remains the primary choice for detecting trypanosomes in blood samples (44). Accordingly, most previous studies in Lambwe were based on microscopic examination of thick blood smears or BCT (13) and could have reported lower prevalence of trypanosomes. The use of PCR as a diagnostic tool has been shown to increase the number of trypanosome infections detected in cattle by at least 2-fold (45–48) and may provide reliable information on prevalence. Lower detection with microscopy relative to the molecular-based technique could result from some sampled cattle at the chronic infection stage when parasitemia is typically lower than an acute clinical stage.

Our result showed that infected cattle were significantly anemic compared to non-infected cattle. Reductions in PCV were due to infection with *T. congolense* but not *T. vivax* and *T. brucei brucei*. We observed that 44% of *T. congolense* infected cattle were anemic (PCV <24%). Anemia is vital in the pathogenesis of trypanosomosis (49) and an indicator of disease severity. The parasite *T. congolense* is known to reduce the PCV or cause anemia in cattle (50). This finding, therefore, suggests that livestock are adversely affected by AAT in the Lambwe Valley and that the majority of severe infections are due to *T. congolense* infections. We also recorded significant rates of anemia in cattle not infected with *T. congolense*, with 24.9% of these recording a mean PCV of <24%. Mixed infection can increase competition for nutrients and space by trypanosomes, thus causing anemia. Though 8.7% of infected animals had mixed infections, we did not find evidence for lowered PCV due to mixed infection level. This result suggests other factors such as helminths, nutrition, and tick-borne diseases. Our other investigation related to this study showed that anaplasmosis also contributed to the anemic state of cattle in the Lambwe Valley (20).

In conclusion, our study demonstrates that control efforts likely eliminated HAT but not bovine trypanosomosis in Lambwe Valley. The risk for AAT remains high around the RNP but is underestimated in previous studies and using microscopy. Molecular diagnostics can aid accurate mapping of trypanosomosis in cattle of Lambwe Valley and enable risk-tailored interventions with potential impact on bovine trypanosomosis.

## Data Availability Statement

The original contributions presented in the study are included in the article/Supplementary Materials, further inquiries can be directed to the corresponding author/s.

## Ethics Statement

The animal study strictly adhered to the experimental guidelines and procedures approved by the Institutional Animal Care and Use Committee at International Centre of Insect Physiology and Ecology (icipe) and Kenya's animal welfare laws under the Veterinary Surgeons and Veterinary Para-professionals Act, 2011 (Cap. 366). Blood samples were collected only after receiving informed verbal consent from cattle keepers. Blood samples were collected by an experienced veterinarian with the aim of minimising pain and discomfort.

## Author Contributions

MO, DM, DG, and SK: conceptualization. PO, MO, KM, and SK: methodology. KM, PO, and SK: formal analysis. PO, MO, FM, EK, DM, and SK: investigation. MO and SK: data curation and writing—original draft preparation. SK, EK, DG, FM, and MO: writing—review and editing. DM and DG: supervision. DM and MO: project administration and funding acquisition. All authors have read and agreed to the published version of the manuscript.

## Funding

This work was supported through the European Union's Integrated Biological Control Applied Research Programme—tsetse repellent component (EU-IBCARP tsetse) awarded to the International Centre of Insect Physiology and Ecology (icipe), and grant number (IBCARP DCI-FOOD/2014/346-739), the German Ministry for Economic Cooperation and Development (B.M.Z.) through the Deutsche Gesellschaft für Internationale Zusammenarbeit (G.I.Z.) ICTDL Project Contract No: 81235250 and Project No: 18.7860.2-001.00 and the DELTAS Africa Initiative grant # DEL-15-011 to THRiVE-The DELTAS Africa Initiative is an independent funding scheme of the African Academy of Sciences (AAS)'s Alliance for Accelerating Excellence in Science in Africa (AESA) and supported by the New Partnership for Africa's Development Planning and Coordinating Agency (NEPAD Agency) with funding from the Wellcome Trust grant # 107742/Z/15/Z. Additionally, we acknowledge institutional financial support to icipe by UK Aid from the UK Government, Swedish International Development Cooperation Agency (Sida), the Swiss Agency for Development and Cooperation (SDC), Federal Democratic Republic of Ethiopia, and the Kenyan Government. The funders had no role in study design, data collection and analysis, design, data collection and analysis, the decision to publish, or manuscript preparation.

## Author Disclaimer

The views expressed in this publication are those of the authors and not necessarily those of any of the funding agencies.

## Conflict of Interest

The authors declare that the research was conducted in the absence of any commercial or financial relationships that could be construed as a potential conflict of interest.

## Publisher's Note

All claims expressed in this article are solely those of the authors and do not necessarily represent those of their affiliated organizations, or those of the publisher, the editors and the reviewers. Any product that may be evaluated in this article, or claim that may be made by its manufacturer, is not guaranteed or endorsed by the publisher.
