# Supplementary material for: Prevalence of Trypanosome Species in Cattle Near Ruma National Park, Lambwe Valley, Kenya: An Update From the Historical Focus for African Trypanosomosis
Source: Front Vet Sci. 2021 Nov 2;8:750169. doi: 10.3389/fvets.2021.750169 (PMC8594777; doi:10.3389/fvets.2021.750169)
Supplement: Supplementary file 1 [file Table_1.DOCX]

**Supplemental table**: Effect of *T. brucei*, *T.congolense*, and *T.vivax* on PCV values. Data from cattle infected with *T. theileri* was excluded due to the smaller sample size.

| Infection | Median PCV (%)±SE |
| --- | --- |
| *T. brucei* | 27.34±0.68 |
| *T. congolense* | 24.95±0.55 |
| *T. vivax* | 27.3±0.64 |
| Mixed infection | 26.96±1.67 |
| Non-infected | 27.74±0.21 |
